# Diagnostic Delay of Celiac Disease in Childhood

**DOI:** 10.1001/jamanetworkopen.2024.5671

**Published:** 2024-04-09

**Authors:** Paola Ilaria Bianchi, Marco Vincenzo Lenti, Clarissa Petrucci, Giulia Gambini, Nicola Aronico, Matteo Varallo, Carlo Maria Rossi, Elena Pozzi, Elena Groppali, Francesca Siccardo, Giulia Franchino, Gian Vincenzo Zuccotti, Grazia Di Leo, Chiara Zanchi, Fernanda Cristofori, Ruggiero Francavilla, Marina Aloi, Giulia Gagliostro, Monica Montuori, Sara Romaggioli, Caterina Strisciuglio, Marco Crocco, Noemi Zampatti, Angela Calvi, Renata Auricchio, Costantino De Giacomo, Silvia Maria Elena Caimmi, Carolina Carraro, Annamaria Staiano, Sabrina Cenni, Mauro Congia, Enrico Schirru, Francesca Ferretti, Carolina Ciacci, Nicoletta Vecchione, Mario Andrea Latorre, Semela Resuli, Giusy Cinzia Moltisanti, Giulia Maria Abruzzese, Andrea Quadrelli, Simone Saglio, Pietro Canu, Damiano Ruggeri, Annalisa De Silvestri, Catherine Klersy, Gian Luigi Marseglia, Gino Roberto Corazza, Antonio Di Sabatino

**Affiliations:** 1 First Department of Internal Medicine, Istituto di Ricovero e Cura a Carattere Scientifico Fondazione Policlinico San Matteo, Pavia, Italy; 2 Department of Internal Medicine and Medical Therapeutics, University of Pavia, Pavia, Italy; 3 Biostatistics and Clinical Trial Center, Fondazione Istituto di Ricovero e Cura a Carattere Scientifico Policlinico San Matteo, Pavia, Italy; 4 Department of Pediatrics, Buzzi Children’s Hospital, Milan, Italy; 5 Department of Pediatrics, Azienda Socio Sanitaria Territoriale Lariana, San Fermo della Battaglia, Como, Italy; 6 Department of Biomedical and Clinical Sciences, University of Milan, Milan, Italy; 7 Institute for Maternal and Child Health, Istituto di Ricovero e Cura a Carattere Scientifico Burlo Garofolo, Trieste, Italy; 8 Interdisciplinary Department of Medicine—Pediatric Section, Aldo Moro University of Bari, Bari, Italy; 9 Pediatric Gastroenterology and Liver Unit, Department of Women’s and Children’s Health, Umberto I Hospital, La Sapienza University, Rome, Italy; 10 Università della Campania Luigi Vanvitelli, Dipartimento della donna, del bambino e della chirurgia generale e specialistica, Naples, Italy; 11 ”Pediatric Gastroenterology and Endoscopy Unit, Istituto di Ricovero e Cura a Carattere Scientifico Giannina Gaslini Institute, Genoa, Italy; 12 Department of Neuroscience, Rehabilitation, Ophthalmology, Genetics, Child and Maternal Health, University of Genoa, Genoa, Italy; 13 Deparment of Translational Medical Sciences, University of Naples Federico II, Naples, Italy; 14 Istituto Europeo per lo Studio delle Malattie correlate ad Alimenti, Naples, Italy; 15 Division of Pediatrics, Department of Mother and Child Health, ASST Grande Ospedale Metropolitano Niguarda, Milan, Italy; 16 Paediatric Clinic, Fondazione Istituto di Ricovero e Cura a Carattere Scientifico Policlinico San Matteo, Pavia, Italy; 17 Department of Clinical, Surgical, Diagnostic and Paediatric Sciences, University of Pavia, Pavia, Italy; 18 Gastroenterologia Pediatrica Clinica Pediatrica e Malattie Rare Ospedale Pediatrico Microcitemico Antonio Cao, Azienda Sanitaria Locale 8, Cagliari, Italy; 19 Centro Servizi di Ateneo per gli Stabulari, Università degli Studi di Cagliari, Cittadella Universitaria, Monserrato, Cagliari, Italy; 20 UO di Gastroenterologia e Riabilitazione Nutrizionale, Ospedale Pediatrico Bambino Gesù, Rome, Italy; 21 University of Salerno Azienda Ospedaliero-Universitaria San Giovanni di Dio e Ruggi d’Aragona of Salerno, Salerno, Italy

## Abstract

**Importance:**

The extent and factors associated with risk of diagnostic delay in pediatric celiac disease (CD) are poorly understood.

**Objectives:**

To investigate the diagnostic delay of CD in childhood, and to assess factors associated with this delay.

**Design, Setting, and Participants:**

Multicenter, retrospective, cross-sectional study (2010-2019) of pediatric (aged 0-18 years) patients with CD from 13 pediatric tertiary referral centers in Italy. Data were analyzed from January to June 2023.

**Main Outcomes and Measures:**

The overall diagnostic delay (ie, the time lapse occurring from the first symptoms or clinical data indicative of CD and the definitive diagnosis), further split into preconsultation and postconsultation diagnostic delay, were described. Univariable and multivariable linear regression models for factors associated with diagnostic delay were fitted. Factors associated with extreme diagnostic delay (ie, 1.5 × 75th percentile) and misdiagnosis were assessed.

**Results:**

A total of 3171 patients with CD were included. The mean (SD) age was 6.2 (3.9) years; 2010 patients (63.4%) were female; and 10 patients (0.3%) were Asian, 41 (1.3%) were Northern African, and 3115 (98.3%) were White. The median (IQR) overall diagnostic delay was 5 (2-11) months, and preconsultation and postconsultation diagnostic delay were 2 (0-6) months and 1 (0-3) month, respectively. The median (IQR) extreme overall diagnostic delay (586 cases [18.5%]) was 11 (5-131) months, and the preconsultation and postconsultation delays were 6 (2-120) and 3 (1-131) months, respectively. Patients who had a first diagnosis when aged less than 3 years (650 patients [20.5%]) showed a shorter diagnostic delay, both overall (median [IQR], 4 [1-7] months for patients aged less than 3 years vs 5 [2-12] months for others) and postconsultation (median [IQR], 1 [0-2] month for patients aged less than 3 years vs 2 [0-4] months for others). A shorter delay was registered in male patients, both overall (median [IQR], 4 [1-10] months for male patients vs 5 [2-12] months for female patients) and preconsultation (median [IQR], 1 [0-6] month for male patients vs 2 [0-6] months for female patients). Family history of CD was associated with lower preconsultation delay (odds ratio [OR], 0.59; 95% CI, 0.47-0.74) and lower overall extreme diagnostic delay (OR, 0.75; 95% CI, 0.56-0.99). Neurological symptoms (78 patients [21.5%]; OR, 1.35; 95% CI, 1.03-1.78), gastroesophageal reflux (9 patients [28.1%]; OR, 1.87; 95% CI, 1.02-3.42), and failure to thrive (215 patients [22.6%]; OR, 1.62; 95% CI, 1.31-2.00) showed a more frequent extreme diagnostic delay. A previous misdiagnosis (124 patients [4.0%]) was more frequently associated with gastroesophageal reflux disease, diarrhea, bloating, abdominal pain, constipation, fatigue, osteopenia, and villous atrophy (Marsh 3 classification).

**Conclusions and Relevance:**

In this cross-sectional study of pediatric CD, the diagnostic delay was rather short. Some factors associated with risk for longer diagnostic delay and misdiagnosis emerged, and these should be addressed in future studies.

## Key Points

**Question:**

Is there a delay in diagnosing pediatric celiac disease (CD) in childhood?

**Findings:**

In this cross-sectional study of 3171 patients in Italy, the median (IQR) diagnostic delay was 5 (2-11) months. In those with less specific symptoms or failure to thrive, the delay was longer, but children younger than 3 years or with a family history of CD were diagnosed earlier.

**Meaning:**

These findings suggest the diagnostic delay of CD is generally low, but some clinical features could be associated with a prolonged or shortened delay.

## Teaser

This cross-sectional study evaluates the diagnostic delay of celiac disease in childhood in Italy, and to assess factors associated with this delay.

## Introduction

Celiac disease (CD) is an immune-mediated, gluten-sensitive enteropathy.^1^ CD has been described worldwide, with a high prevalence in both childhood and adulthood, especially in White people (close to 1%); nonetheless, many patients remain undiagnosed in the real-life setting.^1,2^ Indeed, a strict and lifelong withdrawal from dietary gluten can revert villous atrophy in most cases and may improve quality of life.^3,4^

CD has a wide clinical spectrum^5^ that can make its diagnosis a great challenge, especially in children. In fact, CD in children may be asymptomatic, may cause mild gastrointestinal symptoms, or may present a severe clinical picture, including anemia, failure to thrive or weight loss, dysproteinemia, diarrhea, dehydration, and electrolyte imbalance. This wide heterogeneity may lead to a delayed diagnosis and may also increase the risk of misdiagnosis.^6^ Similar to other immune-mediated gastrointestinal disorders characterized by a wide or unspecific clinical spectrum, such as inflammatory bowel disease^7^ and autoimmune atrophic gastritis,^8^ CD seems to be burdened by a substantial diagnostic delay and a greater risk of mortality in adults.^9,10,11^ Hence, a timely diagnosis of CD is warranted.^3^ However, there are still few data regarding the diagnostic delay in children, as well as possible factors associated with risk, as reported in Table 1, and mostly with a small sample size.^12,13,14,15,16,17,18^ A wide range of diagnostic delay has been reported, reaching up to 10 years in some cases.

**Table 1.** Diagnostic Delay (DD) in Children With Celiac Disease (CD) According to the Available Retrospective Studies

Source	Year(s)	Country	Population	Patients, No.	Data collection	Overall DD, mean (SD)	Median (range)		Main findings
							Preconsultation DD	Postconsultation DD	
Khuffash et al,^12^ 1987	1980-1985	Kuwait	Children	20	Interview	38 mo	NA	NA	Chronic diarrhea is a symptom of CD.
Granot et al^13^ 1994	1978-1988	Israel	Children	65	Interview	Arab children: 25.2 (27) mo; Jewish children: 13.5 (16) mo	NA	NA	Lack of awareness of CD may result in a detrimental DD in specific ethnic groups.
Rashid et al,^14^ 2005	2002	Canada	Children	168	Questionnaire	1 y	NA	NA	Previous misdiagnoses are common; multiple medical consultations before achieving diagnosis are common.
Rodrigo-Saéz et al,^15^ 2011	2000-2006	Spain	Adults and children	144 Adults and 43 children	Medical record	Adults: 10 (9) y; children: 1 (2) y	NA	NA	Classical form of CD is more common in children, in whom DD is lower.
Navalón-Ramon et al,^16^ 2016	1970-2000	Spain	Adults and children	65 Adults and 41 children	Questionnaire	Adults: 7.97 y; children: 0.68 y	NA	NA	Vomiting, anemia, abdominal distension, and failure to thrive are more common in children than adults.
Coppell et al,^17^ 2019	2012	New Zealand	Children <16 y	123	Questionnaire	Median (range), 1.5 (0-11) y	NA	NA	The challenges associated with CD DD in childhood are an important issue.
Riznik et al,^18^ 2019	2017	Croatia, Hungary, Germany, Italy, and Slovenia	Children and adolescents <19 y	393	Medical record	Median (range), 6 mo (0-10) y	5 mo (0-10 y)	1 mo (0-5 y)	DD in children slightly shorter compared with other studies and significantly shorter than in adults.

Abbreviation: NA, not assessed.

The 2012 European Society for Paediatric Gastroenterology Hepatology and Nutrition (ESPGHAN) guidelines recommend using quantitative determination of antitissue transglutaminase IgA (TTG-IgA) as a first-line screening. If the antibody titer is above 10 times the upper limit of normal in a symptomatic patient, the guidelines recommend a second check of TTG-IgA, along with anti-endomysial antibodies (EMA) and human leukocyte antigen (HLA)-DQ2/DQ8 detection. This strategy reduced the need for an upper gastrointestinal endoscopy by 30% to 50%.^19^ In 2020, even less stringent criteria were proposed, also including asymptomatic patients.^20^ Recently, a biopsy-free approach has even been suggested in the diagnosis of adult CD.^21^

Starting from these premises, we hypothesized that: (1) there is still a substantial diagnostic delay for CD; (2) the presenting signs and symptoms can be scarce, tricky, and subtle, thus contributing to the diagnostic delay; and (3) the diagnostic delay in childhood is smaller than the one in adulthood, since in most cases there is no need for a biopsy in the former. Therefore, the main aim of this study was to investigate the diagnostic delay of CD in an Italian multicenter cohort of pediatric patients, highlighting their presenting symptoms, signs, and any concern potentially associated with the delay.

## Methods

### Study Design, Participants, and Collected Variables

This was a retrospective observational multicenter study. At first, 16 Italian gastroenterological, tertiary referral, outpatient pediatric clinics were invited to participate, and 13 of them accepted. The 13 participating centers are located across the country, from Northern to Southern Italy, thus providing a broad overview of pediatric CD in the whole country. The ethics committee of Pavia (Fondazione Istituto di Ricovero e Cura a Carattere Scientifico Policlinico San Matteo) approved the study protocol, and this approval was extended to all the ethics committees of the other participating centers. Either the patient or the caregiver, depending on the age at the time of enrollment, were asked to provide written informed consent to take part in the study. The necessity of a signed consent was waived for those patients who could not be reached, as all data were anonymous. This study followed the Strengthening the Reporting of Observational Studies in Epidemiology (STROBE) reporting guidelines.^22^

The inclusion criteria were as follows: age 18 years or younger, both for male and female patients, and established diagnosis of CD according to the ESPGHAN 2012^19^ guidelines. Conversely, the exclusion criteria were as follows: uncertain or undefined duodenal histological alterations (ie, seronegative duodenal atrophy, unavailable serology, nonatrophic lesions, and TTG-IgA titer less than 10 times the upper limit of normal), noncompatible HLA, absence of relevant data for the study (eg, clear indications of overall, preconsultation, and postconsultation diagnostic delay; sex; age at diagnosis; and year of diagnosis), absence of a follow-up period of at least of 1 year after the diagnosis (ie, only patients who were actually followed up, and hence complete data could be retrievable), diagnosis made in the period of 2020 and 2021 because of the particularity of health care access affected by the COVID-19 pandemic, or diagnosis before 2010 to avoid potential biases due to the unavailability of novel guidelines due to the improvement of CD-specific antibody detection and increasing awareness of CD. Patients in whom the diagnostic delay was unknown could not be included since this was the primary end point of the study. Inclusion and exclusion criteria are summarized in a flowchart in the eFigure in Supplement 1. Self-reported race data were collected in this study because of the possibility of different genetic backgrounds influencing the presentation of CD. Other details regarding patient selection, end points, and statistical analysis are reported in the eMethods in Supplement 1.^23,24,25^

### Statistical Analysis

Univariable and multivariable logistic regression models were fitted using misdiagnoses as dependent variables and sign, symptom, and clinical data as independent variables. Parameters with a *P* value less than .20 at univariable analysis were included in multivariable models. The area under the model receiver operating characteristic curve was computed to assess discrimination. Univariable and multivariable generalized linear regression models (with gaussian family and identity link) were fitted using diagnostic delays as dependent variables and sign, symptom, and clinical data along with clinical characteristics as independent variables. Delays were log-transformed for the purpose of the analysis. Variables with a *P* value less than .20 at univariable analyses were included in the multivariable models. Logistic regression was used to model extreme diagnostic delays; the same variables as for the corresponding delay model were used. Statistical analyses were performed using Stata statistical software, version 17 (StataCorp). A 2-sided *P*-value less than .05 was considered statistically significant. Data were analyzed from January to June 2023.

## Results

### Demographic and Clinical Data

In total, from 3250 patients in the database, 3171 patients were included (mean [SD] age 6.2 [3.9] years; 2010 patients [63.4%] were female; and 10 patients [0.3%] were Asian, 41 patients [1.3%] were Northern African, and 3115 patients [98.3%] were White). A total of 79 patients were excluded as reported in eFigure in Supplement 1. Overall, 1969 patients (62.1%) underwent a duodenal biopsy. CD-specific serology was available in all cases: 3094 (97.6%) had a TTG-IgA quantification, 2586 (81.6%) had EMA quantification, 1013 (31.9%) had an antigliadin antibody (AGA) quantification, and in 1699 (53.6%), HLA-typing was available.

Table 2 reports the demographic characteristics and the clinical manifestations associated with the diagnosis of CD found in the whole cohort. Almost one-third of the patients (903 patients [28.5%]) had more than 2 symptoms. Red blood cell count alterations were, collectively, the most reported alteration (2471 patients [76.7%]), with microcytic anemia being the main one (687 patients [93.3%]). Gastrointestinal symptoms were present in 2119 patients (66.8%).

**Table 2.** Demographic and Clinical Characteristics of the 3171 Patients With Celiac Disease (CD)

Variables	Patients, No. (%)^a^
Age, mean (SD), y	6.16 (3.93)
Age groups, y	
<3	650 (20.50)
3-5	969 (30.56)
6-10	1045 (32.95)
11-18	507 (15.99)
Sex	
Female	2010 (63.39)
Male	1161 (36.61)
Ethnicity	3168 (99.90)
Asian	10 (0.32)
Black	2 (0.06)
Northern African	41 (1.29)
White	3115 (98.33)
No. of siblings	2313 (72.94)
0	810 (35.02)
1	1179 (50.97)
2	256 (11.07)
3	48 (2.08)
4	14 (0.61)
5 or more	5 (0.26)
Male body mass index, median (IQR)^b^	15.64 (14.78-17.15)
Female body mass index, median (IQR)^b^	15.62 (14.61-16.98)
Gastrointestinal symptoms	
≥1 Symptom	2119 (66.82)
>1 Symptom	885 (27.91)
Abdominal pain	1025 (32.32)
Diarrhea	676 (21.32)
Bloating	415 (13.09)
Constipation	396 (12.49)
Weight loss	312 (9.84)
Anorexia	252 (7.95)
Vomiting	214 (6.75)
Dyspepsia	87 (2.74)
GERD	32 (1.01)
Red blood cell count alteration	
≥1	2471/3153 (76.66)
Microcytic anemia	687 (27.8)
Normocytic anemia	5 (4.76)
Macrocytic anemia	6 (0.82)
Neutropenia	4 (0.54)
Thrombocytopenia	3 (0.41)
Pancytopenia	1 (0.14)
Recent gastrointestinal infection	184 (5.84)
Fatigue, No./total No. (%)	436/3166 (13.77)
Associated autoimmune disorders	
≥1	232 (7.32)
>1 Autoimmune disorder	5 (0.16)
Thyroiditis	117 (3.69)
Type 1 diabetes	62 (1.96)
Vitiligo	14 (0.44)
Psoriasis	9 (0.28)
Rheumatoid arthritis	5 (0.16)
Connective tissue disease	3 (0.09)
Addison disease	0
Family history of CD, No./total No. (%)	789/3167 (24.95)
Dental enamel defect, No./total No. (%)	57/3135 (1.82)
Dysproteinemia, No./total No. (%)	55/3145 (1.75)
Osteopenia, No./total No. (%)	44/3004 (1.46)
Failure to thrive, No./total No. (%)	952/3166 (30.07)
Delayed menarche, No./total No. (%)	4/ 312 (1.28)
Neuropsychiatric symptoms	363 (11.45)
Headache	89 (2.81)
Epilepsy	18 (0.57)
Paresthesia	3 (0.09)
Other neuropsychiatric symptoms^c^	253 (7.98)
Dermatitis herpetiformis, No./total No. (%)	66/3167 (2.08)
Selective IgA deficiency, No./total No. (%)	63/3165 (1.99)
Common variable immunodeficiency, No./total No. (%)	8/3167 (0.25)
Down syndrome, No./total No. (%)	6/3161 (0.19)

Abbreviation: GERD, gastroesophageal reflux disease.

^a^ If the number of available data for a variable is different from 3171, it is reported.

^b^ Body mass index is calculated as weight in kilograms divided by height in meters squared.

^c^ These include mood changes, learning disabilities, confusion, memory loss, depression, persecutory delusions, and psychosis.

Interestingly, many differences according to the age group at diagnosis were found (eTable 1 in Supplement 1). Patients who received a first diagnosis when aged less than 3 years (650 patients [20.5%]), compared with all others, had a significantly higher rate of multiple symptoms, gastrointestinal symptoms, weight loss, failure to thrive, dysproteinemia, major presentation of CD, and need for hospitalization. Some differences in the clinical presentation were also found between sexes: the prevalence of autoimmune disorders, a history of CD in a first-grade relative, and the presence of abdominal pain were more frequent in female patients, while diarrhea was more common in male patients. Notably, the rate of nonatrophic duodenal lesions (Corazza-Villanacci 0-A) was, overall, relatively high, with a decreasing trend across the age groups. eTable 2 in Supplement 1 shows the characteristics of patients who were diagnosed with a biopsy compared with those who were not. Notably, the postconsultation (median [IQR] 1 [0-3] month vs 2 [0-4] months) and overall (median [IQR] 4 [1-10] months vs 5 [2-12] months) diagnostic delays were lower in those who did not get a biopsy compared with those who were diagnosed with a biopsy. Also, as shown in eTable 2 in Supplement 1, older patients were more likely to be biopsied, while those with gastrointestinal symptoms were less likely to be biopsied consistently with the guidelines.

### Quantification of the Diagnostic Delay

**Figure 1.**
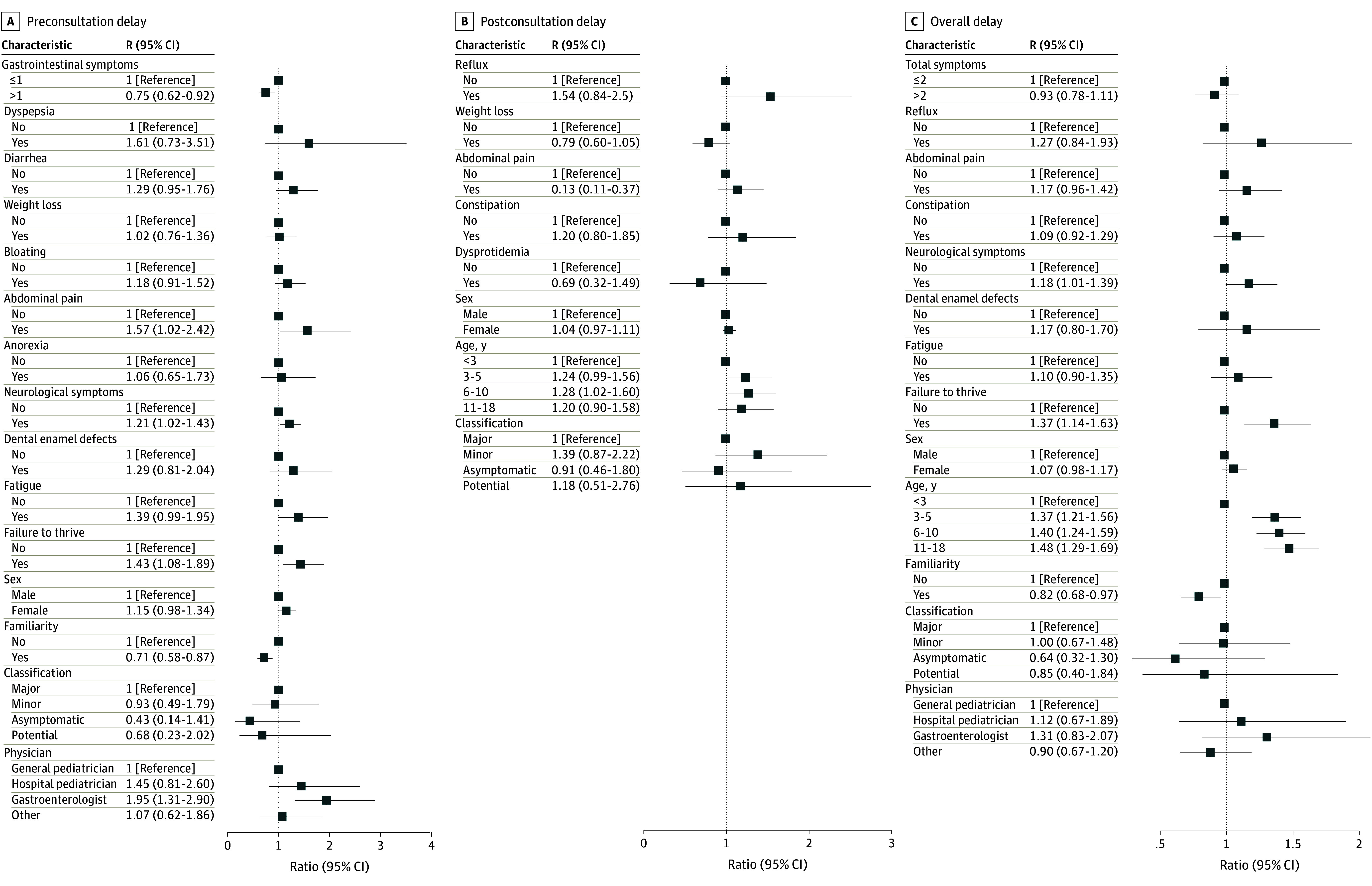
Multivariable Analysis for Factors Associated With Preconsultation, Postconsultation, and Overall Diagnostic Delay For each level of each variable in the model, squares are the estimate of the difference in log delay, back transformed into ratios. Whiskers are 95% CIs. If these do not cross the null effect line, they are significant at the 5% level. R indicates ratio.

The median (IQR) overall diagnostic delay was 5 (2-11) months, while preconsultation and postconsultation diagnostic delays were 2 (0-6) months and 1 (0-3) month, respectively. The overall median (IQR) extreme diagnostic delay was 11 (5-131) months and was present in 555 (17.5%) of the cases, while the preconsultation extreme diagnostic delay was 6 (2-120) months (586 cases [18.5%]), and the postconsultation extreme diagnostic delay was 3 (1-131) months (605 cases [19.1%]). The variables included in the multivariable analysis on the diagnostic delay are reported in Figure 1. Having received a first diagnosis of CD when aged less than 3 years rather than between 3 and 18 years was associated with a lower diagnostic delay, both overall (median [IQR], 4 [1-7] vs 5 [2-12] months; ratio, 0.28; 95% CI, 0.15 to 0.41 for age 3 to 5 years; ratio, 0.32; 95% CI, 0.09 to 0.55 for age 6 to 10 years; ratio, 0.29; 95% CI, 0.07 to 0.52 for age 11 to 18 years) and postconsultation (median [IQR], 1 [0-2] vs 2 [0-4] months; ratio, 0.32; 95% CI, 0.11 to 0.54 for age 3 to 5 years; ratio, 0.39; 95% CI, −0.03 to 0.81 for age 6 to 10 years; ratio, 0.29; 95% CI, −0.16 to 0.73 for age 11 to 18). There were statistically significant differences regarding sex, with overall and preconsultation diagnostic delay being lower in male than in female patients (median [IQR], 4 [1-10] vs 5 [2-12] months; ratio, 0.09; 95% CI, 0.02 to 0.17; and 1 vs 2 months; ratio, 0.19; 95% CI, 0.05 to 0.35, respectively). HLA haplotype, endoscopic appearance, and histologic grade of small-bowel lesions did not affect the timeliness of diagnosis.

### Associations With Extreme Diagnostic Delay

**Figure 2.**
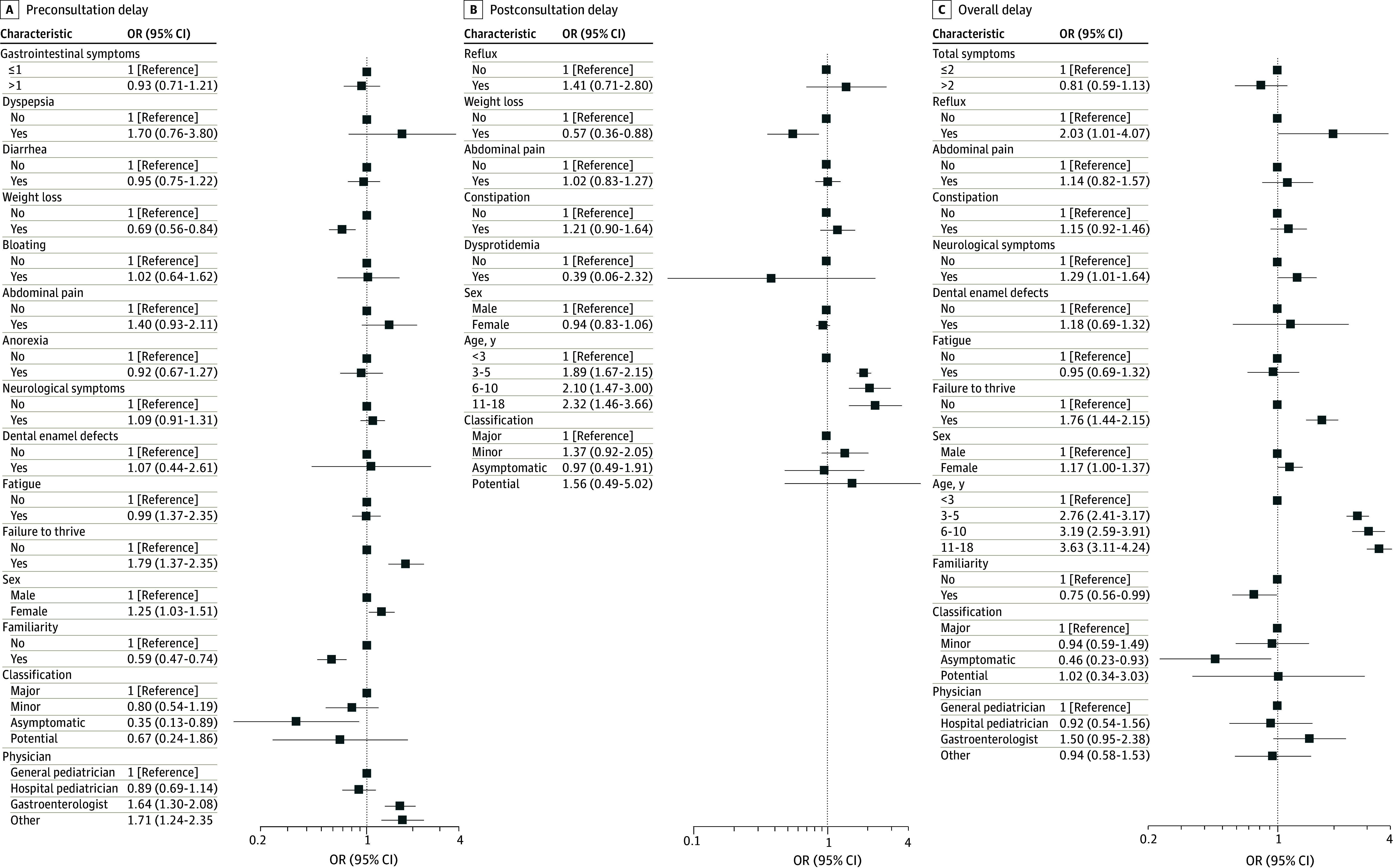
Multivariable Analysis for Factors Associated With the Preconsultation, Postconsultation, and Overall Extreme Delay For each level of each variable in the model, squares are the estimate of the odds ratio (OR). Whiskers are 95% CIs. If these do not cross the null effect line, they are significant at the 5% level.

We observed an extreme diagnostic delay in 587 patients (18.5%). The variables included in the multivariable analysis on the extreme diagnostic delay are reported in Figure 2. The comparators of all variables are reported in eTable 3 in Supplement 1. According to the multivariable analysis, preconsultation extreme diagnostic delay was more common in female patients compared with male patients (400 female patients [19.9%] vs 187 male patients [16.1%]; odds ratio [OR], 1.29; 95% CI, 1.09-1.54), in the case of failure to thrive (246 patients [25.8%]; OR, 1.92; 95% CI, 1.71-2.16), or if the diagnosis was made by a gastroenterologist (86 patients [25.9%]; OR, 1.61; 95% CI, 1.19-2.15) or another nonpediatrician specialist (39 patients [24.5%]; OR, 1.49; 95% CI, 1.07-2.08). A family history of CD (90 patients [11.4%]; OR, 0.59; 95% CI, 0.47-0.74), asymptomatic classification at presentation (33 patients [6.5%]; OR, 0.24; 95% CI, 0.11-0.49), and weight loss (52 patients [16.7%]; 95% CI, 0.87 95% CI, 0.72-1.04) showed a reduced preconsultation extreme diagnostic delay. On the other side, postconsultation extreme diagnostic delay was more common in association with older ages at diagnosis (346 patients [22.3%]; OR, 1.51; 95% CI, 0.98-2.34) and less common in cases of weight loss (33 patients [10.6%]; OR, 0.47; 95% CI, 0.29-0.79) or dysproteinemia (3 patients [5.5%]; OR, 0.24; 95% CI, 0.04-1.51). Conversely, being aged less than 5 years (229 patients [14.14%]; OR, 1.60; 95% CI, 1.40-1.83) and a family history of CD (101 patients [12.8%]; OR, 0.63; 95% CI, 0.48-0.83) were less associated with overall extreme diagnostic delay, while neurological symptoms (78 patients [21.5%]; OR, 1.35; 95% CI, 1.03-1.78), gastroesophageal reflux (9 patients [28.1%]; OR, 1.87; 95% CI, 1.02-3.42), and failure to thrive (215 patients [22.6%]; OR, 1.62; 95% CI, 1.31-2.00) showed a more frequent extreme diagnostic delay. Female patients had a greater extreme overall diagnostic delay (368 patients [18.3%]) compared with male patients, but the difference was not statistically significant (OR, 1.18; 95% CI, 0.99-1.42).

### Misdiagnoses

Finally, the reported misdiagnoses (124 patients [4.0%]) made before the definitive diagnosis of CD were the following: constipation (30 patients), iron deficiency anemia (22 patients), gluten intolerance (without workup for excluding CD; 11 patients), gastroenteritis (9 patients), cow’s-milk protein allergy (9 patients), lactose intolerance (7 patients), food allergy (7 patients), gastroesophageal reflux disease (6 patients), atopic dermatitis (5 patients), poor growing (5 patients), irritable bowel syndrome (3 patients), and others (10 patients). eTable 4 in Supplement 1 summarizes the factors associated with misdiagnoses and the associated univariable and multivariable analysis. At multivariable analysis, gastroesophageal reflux disease, diarrhea, bloating, abdominal pain, constipation, fatigue, osteopenia, and Marsh 3 lesions were all significantly associated with misdiagnosis.

## Discussion

The diagnostic delay of CD in pediatric patients in Italy found in this cross-sectional study seems lower than that reported in the literature. Our study, compared with those already published (Table 1) is currently the largest in the pediatric setting that we know of, and includes a well-characterized cohort of pediatric patients with CD. We have herein reported the diagnoses made after the introduction of the biopsy-sparing ESPGHAN guidelines of 2012. Moreover, this is one of the few studies that distinguishes preconsultation and postconsultation delay and focuses on factors associated with cases of extreme diagnostic delay. Our results confirm and corroborate previous findings on major symptoms as they frequently present in childhood. Also, the biopsy-sparing approach was found to be burdened by a lower overall delay, possibly due to time saved by making an appointment for the upper gastrointestinal endoscopy and for the histopathological report.

In fact, the diagnostic delay of CD in children is here confirmed^18^ to be shorter than in adults by comparison with data from a national multicenter study by our group^11^ that includes the same period of years and a similar method of data description. In the adult study, overall, preconsultation, and postconsultation diagnostic delays were 8, 3, and 4 months, respectively, compared with 5, 2, and 1 in the pediatric study. The diagnosis of CD in children is usually simpler because the general pediatrician and the hospital pediatrician would lead the process and the patient does not usually consult a series of other specialists for different symptoms. A broad adherence to the international guidelines for the diagnosis of CD should probably be considered another important element of this success. The biopsy-sparing policy was likely advantageous in lowering the mean time to diagnose CD, as the time needed for making the appointment and analyzing the biopsy is completely absent. The counterpart of the application of ESPGHAN guidelines is that a retrospective study based on clinical records, such as the present one, would be lacking many theoretically interesting data, such as HLA-typing, EMA and AGA levels, endoscopic findings, and histological grading.

Given the shortness of diagnostic delay found in this study, we consider the results regarding extreme diagnostic delays more important. Moreover, there are some factors that affect diagnostic delay of CD both in adults and in children, such as a previous misdiagnosis.^11,26^ The rate of false diagnoses before CD diagnosis is lower in the pediatric population as compared with the adult population. Luckily, the diagnostic delay of CD in children is not burdened by the risk of typical complications of CD (ie, refractoriness and enteropathy-associated T-cell lymphoma), but can imply a severe malabsorption syndrome anyway, with the need for hospitalization and parenteral nutrition.

This study, for its magnitude, has the merit of depicting the current clinical presentation of Italian pediatric patients with CD. The fairly high rate of detection of nonatrophic lesions in the group of patients who underwent endoscopy is similar to those reported from a review of the literature.^20^ There are also some clinical similarities in the presentation of CD in adult and pediatric Italian populations, such as a very high prevalence of red blood cell count alterations and particularly microcytic anemia and gastrointestinal symptoms. The prevalence of autoimmune diseases seems to be lower in the pediatric population compared with adults, as expected, and this tended to be associated with shorter diagnostic delay, though at the limit of significance. This result may suggest the need for enforcing screening in patients with multiple autoimmune comorbidities. A family history of CD and an asymptomatic presentation were associated with shorter delays in both age ranges, because patients with these characteristics are likely to have been diagnosed by a case-finding strategy (due to the family history or the presence of conditions associated with CD).

A very interesting finding is that younger patients reached the diagnosis in less time, especially patients younger than 3 years for whom extreme diagnostic delay was rare. These data confirm a similar trend observed in another recent European study^19^ and would probably have been an unexpected result in the past, when serological tests were not as reliable as they are today.

The higher prevalence of failure to thrive in patients who were burdened by a higher diagnostic delay may have different explanations. Failure to thrive could be attributed to diseases other than CD, and milder cases could be overlooked. Notably, it has already been reported that the presence of a classical malabsorption syndrome in adult patients was associated with a longer delay.^9,26^ Given the fact that the delay depends mostly on patients, this means that some patients and their families may delay seeking medical care. It should be stressed that in Italy there is universal coverage of health care expenses, which can also be full coverage in cases of medium to low family income, so pediatric treatments are universally available to anyone. In other countries, priority in the health care system may depend on or be influenced by the economic status of the individual, and hence our results may not be applicable to other settings.

The difference found between sexes on overall and preconsultation delays did not seem clinically relevant (5 vs 4 months and 2 vs 1 month). On the other hand, it is easy to hypothesize that gastroesophageal reflux or other signs or disease associations, all associated with a longer diagnostic delay, can be initially misinterpreted and prompt the assessment of specialists other than pediatricians, with a consequently longer diagnostic process. It is notable that osteopenia, which is more typically related to adult CD, was reported in 44 cases. Indeed, older children are more likely to get a workup for osteopenia than younger children, and some rare cases of osteopenia in infants could be missed.

### Limitations

Our study has some limitations that must be mentioned. First, the retrospective nature and telephone interview are open to some biases; for example, the date of symptom onset may be inaccurate, and some physician consultations could have been missed. For some variables, the percentage of missing data was too high, so they were removed, such as those regarding socioeconomic aspects. Also, since White individuals were the most represented, our data cannot be generalized to other ethnicities. The generalizability of our results is also limited to those countries adhering to the ESPGHAN guidelines. Indeed, the setting is tertiary referral outpatient clinics, and therefore these findings cannot address primary care practice. A subanalysis depending on the actual modality of diagnosis could not be made either, as this would have introduced substantial biases, given that the precise reason for the endoscopy and the single diagnostic process could not be clearly retrieved in all cases; this was outside the scope of this research. The latest 2020 ESPGHAN guidelines were not applied here since patients diagnosed after 2020 were not included due to the COVID-19 pandemic. Additionally, we are aware that both the preconsultation and postconsultation delays could be affected by the time needed for making an appointment or other system-level factors that could not be captured. Regardless, this is still the largest study we know of looking at diagnostic delay in pediatric CD, in which a thorough analysis was made and patients were enrolled from almost all Italian regions, thus providing a global picture of the entire country.

## Conclusions

In this cross-sectional study of pediatric CD, the diagnosis was usually made in a timely fashion, except for some cases in which both gastrointestinal and less common symptoms or disease associations led to a prior misdiagnosis and diagnostic delay. A better awareness of pediatric CD proteiform manifestations could be helpful in early recognition of CD.

## Notes

Supplement 1. **eFigure.** Pediatric Celiac Disease Diagnostic Delay: Flow Chart of the Inclusion and Exclusion Criteria of the Patients
eMethods.
**eTable 1.** Characteristics According to Different Age Groups **eTable 2.** Characteristics of Patients Who Were Diagnosed With a Biopsy Compared With Those Who Were Not **eTable 3.** Univariable Analyses for Variables Studied in Extreme Diagnostic Delay of Pediatric CD **eTable 4.** Univariable and Multivariable Analyses for Factors Associated With Misdiagnosis Previous to Celiac Disease Diagnosis

Supplement 2.
Data Sharing Statement
