# Supplementary material for: Diagnostic Delay of Celiac Disease in Childhood
Source: JAMA Netw Open. 2024 Apr 9;7(4):e245671. doi: 10.1001/jamanetworkopen.2024.5671 (PMC11004829; doi:10.1001/jamanetworkopen.2024.5671)
Supplement: Supplement 1. — eFigure. Pediatric Celiac Disease Diagnostic Delay: Flow Chart of the Inclusion and Exclusion Criteria of the Patients eMethods. eTable 1. Characteristics According to Different Age Groups eTable 2. Characteristics of Patients Who Were Diagnosed With a Biopsy Compared With Those Who Were Not eTable 3. Univariable Analyses for Variables Studied in Extreme Diagnostic Delay of Pediatric CD eTable 4. Univariable and Multivariable Analyses for Factors Associated With Misdiagnosis Previous to Celiac Disease Diagnosis [file jamanetwopen-e245671-s001.pdf]

Supplemental Online Content

Bianchi PI, Lenti MV, Petrucci C, et al. Diagnostic delay of celiac disease in childhood. *JAMA Netw Open*. 2024;7(4):e245671. doi:10.1001/jamanetworkopen.2024.5671

eFigure. Pediatric Celiac Disease Diagnostic Delay: Flow Chart of the Inclusion and Exclusion Criteria of the Patients
eMethods
eTable 1. Characteristics According to Different Age Groups
eTable 2. Characteristics of Patients Who Were Diagnosed With a Biopsy Compared With Those Who Were Not
eTable 3. Univariable Analyses for Variables Studied in Extreme Diagnostic Delay of Pediatric CD
eTable 4. Univariable and Multivariable Analyses for Factors Associated With Misdiagnosis Previous to Celiac Disease Diagnosis

This supplemental material has been provided by the authors to give readers additional information about their work.

© 2024 Bianchi PI et al. *JAMA Network Open.*

eFigure 1. Pediatric celiac disease diagnostic delay: Flow chart of the inclusion and exclusion criteria of the patients.

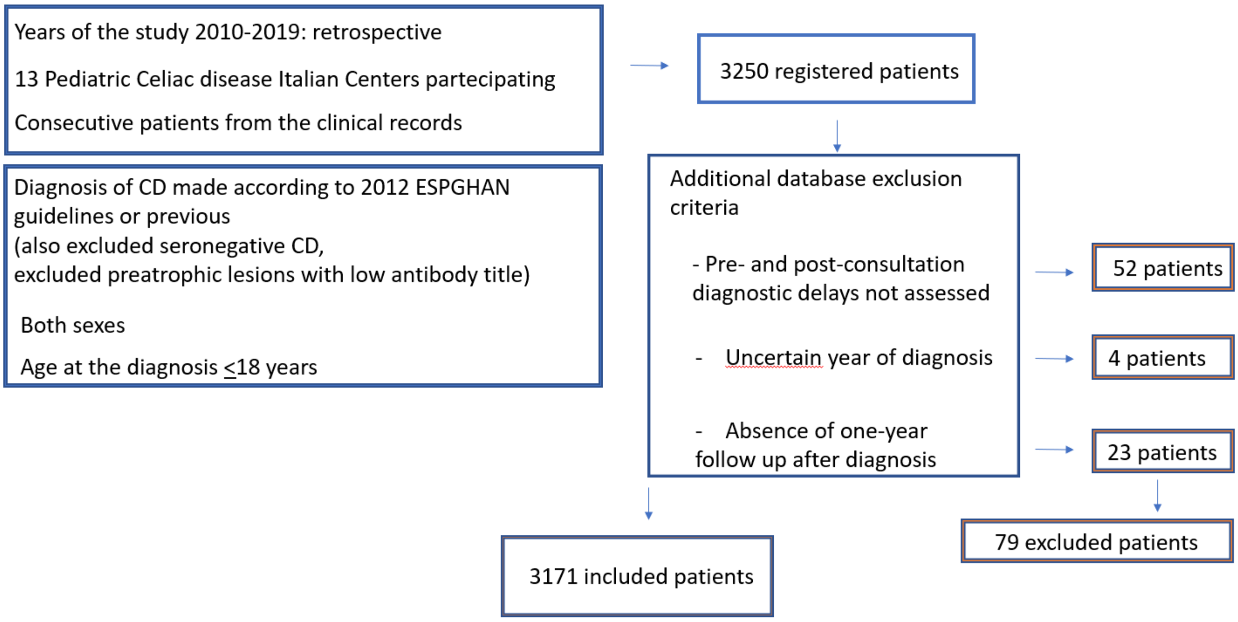

© 2024 Bianchi PI et al. *JAMA Network Open.*

eMethods

Patients' data were retrieved from the local electronic records of each participant center and transferred onto a pseudo-anonymized electronic record form. A telephone interview was also conducted to retrieve missing data, by using a standardized spreadsheet with all the variables to be collected. Each center pseudo-anonymized the data by their own numerical key. All the electronic record forms were merged and reviewed by the study coordinators (center of Pavia). The variables to be included were initially chosen by the study coordinators, and then agreed upon by all study participants. Per each center, a senior pediatrician has been involved, along with two to three consultant pediatricians. All the staff involved were expert in the diagnosis and management of CD. Hence all charts were reviewed by expert physicians, and all variables had been clearly categorized so to avoid biases. When the exact day of symptom onset was not known, this was approximated to the month of onset.

The variables collected included sociodemographic data (i.e., sex, age, ethnicity, number of siblings), clinical variables (e.g., body mass index), medical history, and a wide variety of signs or symptoms of CD (i.e., gastrointestinal symptoms, red blood cell count alterations, fatigue, associated autoimmune disorders, family history of CD, dental enamel defects, dysproteinemia, osteopenia, failure-to-thrive, delayed menarche, neuropsychiatric symptoms, dermatitis herpetiformis, selective IgA deficiency, common variable immune deficiency, Down's syndrome). The ethnicity was defined by the investigators and categorized into Asian, Black, Northern African, White. All misdiagnoses formally made by a physician made before the definitive diagnosis of CD were also recorded. When available, the duodenal histopathological lesions were reported by using the Marsh-Oberhuber classification (i.e., no lesions, Mars 0; intraepithelial lymphocytosis only, Marsh 1; crypt hyperplasia, Marsh 2; villous atrophy, Marsh 3) or the Corazza-Villanacci classification (i.e.,

© 2024 Bianchi PI et al. *JAMA Network Open.*

non-atrophic lesions, A; Mild atrophy, B1; severe atrophy, B2).[22] The Biagi's score for assessing the adherence to a gluten free diet was also reported when available.[23]

The ethics committee of Pavia (Fondazione IRCCS Policlinico San Matteo) approved the study protocol (June 13th 2022) and this approval was extended to all the ethics committees of the other participating centers. Either the patient or the caregiver, depending on the age at the time of enrollment, were asked to sign a written informed consent to take part in the study. The necessity of a signed consent was waived for those patients who could not be reached, as all data were anonymous.

All the findings of the study conform to the STROBE standards for quality control.[24] The raw data of the study cannot be made publicly available due to privacy restrictions but can be shared by the corresponding author upon reasonable request.

Search strategy for literature review

We searched PubMed in September 2023, for all articles in English published since inception, using the medical subject heading terms "celiac disease" or "coeliac disease" AND "diagnostic delay", "children", "pediatric". A total of 253 entries were found with this search strategy. Two authors (MVL, PIB) reviewed all articles and included those focusing on diagnostic delay in the pediatric setting.

Endpoints of statistical analyses and outcome definition

The primary endpoint of the study was to determine the overall diagnostic delay, defined as the time interval between the first likely symptoms of CD, according to the available literature, and the definitive CD diagnosis. Diagnostic delay was distinguished in pre-consultation delay (i.e., the time span between the onset of symptoms and the first medical consultation) and post-consultation delay (i.e., the time span between the first medical

© 2024 Bianchi PI et al. *JAMA Network Open.*

consultation and the definitive diagnosis). By first medical consultation we meant any assessment due to sign/symptom directly related to CD or other related clinical data (e.g., occurrence of autoimmune diseases associated with CD, other conditions related to CD, family history of CD). Assessments made in the primary care setting were also taken into account. Additionally, the secondary endpoints of the study were to describe all the possible presenting signs, symptoms, and clinical data (descriptive analysis), and looking at whether some of them were related to a greater delay or to a misdiagnosis.

Statistical analysis

To reach the endpoints, we retrieved data from 3000 patients. With this sample size, we were able to compute a 95% confidence interval (CI) for the median. We based this calculation as recommended on a paper by Campbell & Gardner[25], where observations are ranked, and the rank is used for the estimation of the 95% CI in our cohort; this extends from 1446 to 1555 which is sufficiently narrow. We used median and interquartile range (IQR, i.e., 25th-75th percentiles) or mean and standard deviation (SD), in case of normal distribution, to describe continuous data, while categorical data were described with counts and percentage. Percentages were calculated after the exclusion of patients with missing data when this was the case. All non-critical variables with data lacking in a percentage higher than 20% were not considered for the statistical analysis. Patients' characteristics were compared between age groups using the Cuzick's test for trend. Median overall diagnostic delay is presented in months with 25th-75th percentiles. Extreme diagnostic delay -overall, pre-consultation, and post-consultation- was calculated as the subgroups of patients with a diagnostic delay of more than 1.5 times the 75th percentile.

As potential clinical data indicative of CD, we included a first-grade family history of CD, the presence of autoimmune comorbidities, dermatitis herpetiformis, selective IgA deficiency, common variable immune deficiency, and Down's syndrome. The patients were classified

© 2024 Bianchi PI et al. *JAMA Network Open.*

according to the clinical presentation as having major (i.e., malabsorption syndrome, with diarrhea, weight loss, failure-to-thrive, nutrient and micronutrients deficiencies), minor (e.g., only isolated symptoms and single alterations, such as isolated iron deficiency anemia, unexplained osteopenia), and asymptomatic CD (i.e., completely asymptomatic and with no laboratory alterations). Potential CD was defined in case of patients with positivity to CD-specific antibodies, but absence of intestinal villous atrophy, when biopsies were taken. As per guidelines, asymptomatic patients were diagnosed by biopsy.

Univariable and multivariable logistic regression models were fitted using misdiagnoses as dependent variables and sign/symptoms/clinical data as independent variables. Parameters with p<0.20 at univariable analysis were included in multivariable models. The area under the model ROC curve was computed to assess discrimination. Univariable and multivariable generalized linear regression models (with gaussian family and identity link) were fitted using diagnostic delays as dependent variables and sign/symptoms/clinical data, and clinical characteristics as independent variables. Delays were log-transformed for the purpose of the analysis. Variables with p<0.20 at univariable analyses were included in the multivariable models. Logistic regression was used to model extreme diagnostic delays; the same variables as for the corresponding delay model were used. The statistical analyses were performed by using STATA statistical software, release 17 (StataCorp, College Station, TX). A two-sided p-value <0.05 was considered statistically significant.

© 2024 Bianchi PI et al. *JAMA Network Open.*

eTable 1. Characteristics According to Different Age Groups

Variable	*< 3 years*	*3-5 years*	*6-10 years*	*11-18 years*	*p (for trend)*
Males	230 (19.81)	358 (30.84)	379 (32.64)	194 (16.71)	0.21
Females	420 (20.90)	611 (30.40)	666 (33.13)	313 (15.57)	0.53
BMI	15.75 (1.56)	15.49 (1.48)	15.89 (1.93)	17.78 (2.07)	0.001
**Hospitalization**	57 (8.77)	42 (4.33)	31 (2.97)	20 (3.94)	0.001
**Number of diagnostic clues**	1.09 (0.28)	1.08 (0.27)	1.06 (0.25)	1.04 (0.19)	0.74
**White ethnicity, n (%)**	639 (98.31)	935 (96.59)	1.018 (97.51)	*501 (99.01)*	0.26
**Multiple symptoms**	254 (39.08)	263 (27.14)	267 (25.55)	118 (23.27)	<0.001
**Sum of symptoms**	2.37 (1.59)	1.85 (1.45)	1.78 (1.38)	1.70 (1.45)	<0.001
**Number of g.i. symptoms**	1.44 (1.22)	1.01 (1.03)	1.04 (1.02)	0.90 (1.03)	<0.001
**Gastrointestinal symptoms (at least 1), n (%)**					
>1 symptom	255 (39.23)	246 (25.39)	275 (26.32)	109 (21.50)	<0.001
Abdominal pain	112 (17.23)	289 (29.82)	439 942.01)	185 (36.49)	<0.001
Diarrhea	286 (44.00)	167 (17.23)	139 (13.30)	84 (16.57)	<0.001
Bloating	136 (20.92)	131 (13.52)	107 (10.24)	41 (8.09)	<0.001
Constipation	80 (12.31)	134 (13.83)	143 (13.68)	39 (7.69)	0.002
Weight loss	120 (18.46)	83 (8.57)	75 (7.18)	34 (6.71)	<0.001
Anorexia	75 (11.54)	86 (8.88)	73 (6.99)	18 (3.55)	<0.001
Vomiting	92 (14.15)	55 (5.68)	46 (4.40)	21 (4.14)	<0.001
Dyspepsia	24 (3.69)	16 (1.65)	29 (2.78)	18 (3.55)	0.04
GERD	6 (0.92)	6 (0.62)	14 (1.34)	6 (1.18)	0.40
**Red blood cell count alteration (at least 1), n (%)**	161 (25.00)	280 (29.02)	192 (18.50)	103 (20.36)	<0.001
Microcytic anemia	147 (91.30)	262 (93.57)	181 (94.27)	97 (94.17)	
Normocytic anemia	12 (7.45)	14 (5.00)	6 (3.13)	3 (2.91)	
Macrocytic anemia	0 (0.00)	2 (0.71)	2 (1.04)	2 (1.94)	
Neutropenia	0 (0.00)	1 (0.36)	2 (1.04)	1 (0.97)	
Thrombocytopenia	2 (1.24)	1 (0.36)	0 (0.00)	0 (0.00)	
**Recent gastrointestinal infection, n (%)**	71 (10.92)	54 (5.63)	40 (3.85)	19 (10.78)	<0.001
**Fatigue**	62 (9.57)	114 (11.76)	146 (14.00)	114 (22.53)	<0.001
**Associated autoimmune disorders (at least 1), n (%)**	37 (5.75)	52 (5.39)	82 (7.88)	61 (12.03)	<0.001
**Family history of CD**	140 (21.64)	260 (26.92)	262 (25.10)	127 (25.15)	0.12
**Dental enamel defect**	3 (0.47)	11 (1.15)	24 (2.32)	19 (3.78)	<0.001
**Dysproteinemia**	38 (5.89)	9 (0.94)	6 (0.58)	2 (0.40)	<0.001
**Osteopenia**	0 (0.00)	7 (0.77)	16 (1.60)	21 (4.25)	<0.001
**Failure-to-thrive**	271 (41.82)	333 (34.40)	275 (26.37)	73 (14.40)	<0.001
**Neuropsychiatric symptoms**	72 (11.11)	75 (7.77)	132 (12.66)	84 (16.60)	<0.001
Headache	4 (80.00)	8 (80.00)	50 (98.04)	27 (87.10)	<0.03
**Dermatitis herpetiformis**	10 (1.54)	21 (2.17)	21 (2.01)	14 (2.78)	0.53
**Selective IgA deficiency**	28 (4.31)	15 (1.55)	14 (1.34)	6 (1.19)	<0.001
**Common variable immunodeficiency**	5 (0.77)	1 (0.10)	2 (0.19)	0 (0.00)	0.047
**Down's syndrome**	1 (0.15)	1 (0.10)	3 (0.29)	1 (0.20)	0.94
**Biagi's GFD adherence score (1-4): mean (sd)**	3.67 (0.59)	3.74 (0.50)	3.67 (0.55)	3.62 (0.65)	0.68
**Diagnosis made by screening**	67 (10.31)	164 (16.92)	201 (19.23)	115 (22.68)	<0.001

© 2024 Bianchi PI et al. *JAMA Network Open.*

**Diagnosis made for clinical evidence**	528 (81.23)	657 (67.80)	704 (67.37)	304 (59.96)	<0.001
**Previous misdiagnosis**	20 (3.19)	40 (4.25)	40 (3.93)	24 (4.84)	0.54
**HLA DQ2 homozygosis**	123 (32.54)	184 (32.97)	188 (35.01)	85 (37.61)	0.48
**TTG IgA title > 10x ULN**	507 (79.84)	674 (71.02)	654 (64.24)	305 (61.99)	<0.001
**EMA positivity**	503 (94.91)	745 (95.27)	815 (94.33)	386 (94.76)	0.79
**AGA IgG title > 10x ULN**	144 (48.81)	162 (51.76)	165 (58.72)	64 (51.61)	0.11
**Endoscopic signs of CD**	130 (78.78)	268 (79.76)	436 (78.82)	179 (77.83)	0.35
**Corazza-Villanacci A (pre-atrophic histological lesions)**	26 (10.28)	45 (8.38)	41 (5.85)	22 (6.06)	0.005
**Clinical classification**					<0.001
Major	433 (67.45)	475 (49.63)	436 (42.21)	206 (40.95)	
Minor	134 (20.87)	296 (30.93)	378 (36.59)	171 (34.00)	
Asymptomatic	55 (8.57)	153 (15.99)	191 (18.49)	109 (21.67)	
Potential	20 (3.12)	33 (3.45)	28 (2.71)	17 (3.38)	
**Physician who made the diagnosis**					<0.001
General pediatrician	204 (31.38)	321 (33.13)	365 (34.93)	122 (24.06)	
Hospital pediatrician	372 (22.30)	528 (54.65)	526 (50.33)	242 (47.73)	
Gastroenterologist	55 (16.57)	84 (8.67)	119 (11.39)	74 (14.60)	
Other specialist	19 (11.95)	36 (22.64)	35 (22.01)	69 (43.40)	
**Pre-consultation delay**	3.37 (4.67)	5.16 (8.12)	5.98 (11.13)	6.38 (11.80)	0.52
**Post-consultation delay**	1.73 (2.46)	3.13 (5.27)	3.48 (6.43)	3.46 (8.14)	<0.001
**Overall diagnostic delay**	5.10 (5.41)	8.27 (9.73)	9.46 (13.28)	9.84 (14.71)	<0.001

Abbreviations: AGA, anti-deamidated gliadin antibodies; CD, celiac disease; EMA, anti-endomysial antibodies;GI, gastrointestinal; GERD, gastro-esophageal reflux disease; GFD, gluten-free diet: HLA, human leukocyte antigen; n.s., non-significative; SD, standard deviation, TTG, anti-transglutaminase antibodies; ULN, upper limit of normal.

© 2024 Bianchi PI et al. *JAMA Network Open.*

eTable 2. Characteristics of Patients Who Were Diagnosed With a Biopsy Compared to those Who Were Not

		Biopsy performed	Biopsy not performed	Prob > |z|	Fisher's exact p
**Total N (%)**		1969 (62.1)	1202 (37.9)		
**Diagnostic delay: median (IQR) (months)**					
Overall		5 (2-12)	4 (1-10)	<0.001	
Pre-consultation		2 (0-6)	2 (0-6)	0.19	
Post-consultation		2 (0-4)	1 (0-3)	<0.001	
**BMI, mean (SD)**		16.02 (1.88)	15.88 (1.87)	0.08	
**Sex**					0.24
Males (1161)		705 (71.7)	456 (39.3)		
Females (2010)		1264 (62.9)	746 (37.1)		
**Age at diagnosis (years)**					< 0.001
<3 N (%)		289 (44.5)	361 (55.5)		
3-5 N (%)		581 (60.0)	388 (40.0)		
6-10 N (%)		734 (70.2)	311 (29.8)		
11-18 N (%)		365 (72.0)	142 (28.0)		
**Family history of CD**	Absent	1470 (61.9)	903 (38.1)		0.53
	Present	499 (63.2)	290 (36.8)		
**Autoimmune diseases**	Absent	1814 (62.0)	110 (38.0)		0.62
	Present	148 (63.8)	84 (36.2)		
**Multiple symptoms**	Absent	1510 (66.5)	759 (33.5)		<0.001
	Present	459 (50.9)	443 (49.1)		
**Multiple diagnostic clues**	Absent	1844 (63.9)	1042 (36.1)		<0.001
	Present	95 (43.6)	123 (56.4)		
**Multiple GI symptoms**	Absent	1524 (66.7)	762 (33.3)		<0.001
	Present	445 (50.3)	440 (49.7)		
**Blood cell count alterations**	Absent	1529 (63.3)	888 (36.7)		0.04
	Present	434 (58.97)	302 (41.0)		
**Dyspepsia**	Absent	1911 (62.0)	1173 (38.0)		0.43
	Present	58 (66.7)	29 (33.3)		
**GERD**	Absent	1943 (61.9)	1196 (38.1)		0.03
	Present	26 (81.3)	6 (18.8)		
**Diarrhea**	Absent	1654 (66.3)	841 (33.1)		<0.001
	Present	315 (46.6)	361 (53.4)		
**Weight loss**	Absent	1836 (64.2)	1023 (35.8)		<0.001
	Present	133 (42.6)	179 (57.4)		
**Vomiting**	Absent	1870 (63.2)	1087 (36.8)		<0.001
	Present	99 (46.3)	115 (53.7)		
**Bloating**	Absent	1760 (63.9)	996 (36.1)		<0.001
	Present	209 (50.4)	206 (49.6)		
**Abdominal pain**	Absent	1330 (62.0)	816 (38.0)		0.88
	Present	639 (62.3)	386 (37.7)		

© 2024 Bianchi PI et al. *JAMA Network Open.*

**Anorexia**	Absent	1836 (62.9)	1083 (37.1)		0.002
	Present	133 (52.8)	119 (47.2)		
**Constipation**	Absent	1738 (62.6)	1037 (37.4)		0.11
	Present	231 (58.3)	165 (41.7)		
**Neurological symptoms**	Absent	1741 (62.2)	1058 (37.8)		0.73
	Present	222 (61.2)	141 (38.8)		
**Dysproteinemia**	Absent	1927 (62.4)	1163 (37.6)		0.09
	Present	28 (50.9)	27 (49.1)		
**Fatigue**	Absent	1692 (62.0)	1038 (38.0)		0.75
	Present	274 (62.8)	162 (37.2)		
**Osteopenia**	Absent	1840 (62.2)	1120 (37.8)		0.88
	Present	28 (63.6)	16 (36.4)		
**Failure-to-thrive**	Absent	1480 (66.8)	734 (33.2)		<0.001
	Present	487 (51.2)	465 (48.8)		
**TTG title < 10x ULN**		812 (85.1)	142 (14.9)		<0.001
**TTG title ≥ 10x ULN**		1095 (51.2)	1045 (48.8)		
**Diagnosis made by**					
- screening		396 (72.4)	151 (27.6)		
- clinical evidence		1276 (58.1)	917 (41.8)		
**Clinical classification**					<0.001
Major		796 (51.4)	754 (48.7))		
Minor		681 (69.6)	298 (30.4)		
Asymptomatic		391 (77.0)	117 (23.0)		
**Physician who made the diagnosis**					0.021
General pediatrician		614 (60.7)	398 (39.3)		
Hospital pediatrician		1065 (63.8)	603 (36.2)	0.66	
Gastroenterologist		185 (55.7)	147 (44.3)	0.001	
Other specialist		105 (66.0)	54 (34.0)	0.018	

Abbreviations: CD, celiac disease; GERD, gastro-esophageal reflux disease; GI, gastrointestinal; IQR, interquartile range; N, number; SD, standard deviation; TTG, anti-tissue transglutaminase antibodies; ULN, upper limit of normal.

© 2024 Bianchi PI et al. *JAMA Network Open.*

eTable 3. Univariable Analyses for Variables Studied in Extreme Diagnostic Delay of Pediatric CD

	Extreme overall DD, n (%)	Univariable analysis OR (95% CI)	p-value	Extreme pre-consulting DD, n (%)	Univariable analysis OR (95% CI)	p-value	Extreme post-consulting DD, n (%)	Univariable analysis OR (95% CI)	p-value
**Sex**			0.07			0.004			0.31
**Males**	185 (15.96)	1.000		187 (16.13)	1.000		229 (19.76)	1.000	
**Females**	368 (18.34)	1.182 (0.985-1.418)		400 (19.93)	1.294 (1.088-1.539)		374 (18.63)	0.930 (0.808-1.071)	
**Age at diagnosis (years)**									
**<3**	52 (8.00)	1.000		69 (10.62)	1.000		68 (10.46)	1.000	
**3-5**	177 (18.27)	2.570 (1.969-3.354)	< 0.001	188 (19.40)	2.027 (1.737-2.365)	< 0.001	190 (19.61)	2.088 (1.888-2.308)	< 0.001
**6-10**	216 (20.67)	2.996 (2.155-4.166)	< 0.001	216 (20.67)	2.194 (1.873-2.570)	< 0.001	229 (21.91)	2.402 (1.542-3.741)	< 0.001
**11-18**	108 (21.30)	3.113 (2.415-4.011)	< 0.001	114 (22.49)	2.443 (2.081-2.867)	< 0.001	117 (23.08)	2.568 (1.538-4.286)	< 0.001
**Family history of CD**	101 (12.80)	0.627 (0.475-0.829)	0.001	90 (11.41)	0.488 (0.378-0.632)	<0.001	139 (17.62)	0.880 (0.630-1.229)	0.45
**Multiple symptoms**	169 (18.74)	1.132 (0.776-1.651)	0.52	190 (21.06)	1.258 (1.001-1.582)	0.049	139 (15.41)	0.707 (-0.460-1.087)	0.11
**Multiple diagnostic clues**	36 (16.51)	0.928 (0.524-1.643)	0.80	36 (16.51)	0.859 (0.488-1.514)	0.60	55 (25.23)	1.476 (-0.750-2.904)	0.26
**Multiple GI symptoms**	152 (17.18)	0.975 (0.778-1.222)	0.83	178 (20.11)	1.155 (0.918-	0.22	151 (17.06)	0.832 (-0.642-	0.17

© 2024 Bianchi PI et al. *JAMA Network Open.*

					1.454)			1.079)	
**Blood cell count alterations**	127 (17.26)	0.977 (0.725-1.318)	0.88	138 (18.75)	1.014 (0.753-1.366)	0.93	127 (17.26)	0.853 (0.608-1.195)	0.35
**GI symptoms (at least one)**	404 (19.07)	1.420 (1.047-1.925)	0.02	463 (21.85)	2.081 (1.610-2.690)	<0.001	388 (18.31)	0.867 (0.604-1.246)	0.44
**Dyspepsia**	23 (26.44)	1.732 (0.748-4.099)	0.20	24 (27.59)	1.706 (0.819-3.555)	0.15	16 (18.39)	0.957 (0.421-2.172)	0.92
**GERD**	9 (28.13)	1.867 (1.019-3.420)	0.04	8 (25.00)	1.474 (0.939-2.313)	0.09	8 (25.00)	1.422 (0.763-2.650)	0.27
**Diarrhea**	108 (15.98)	0.876 (0.715-1.072)	0.20	133 (19.67)	1.101 (0.796-1.523)	0.56	108 (15.98)	0.766 (0.502-1.169)	0.22
**Weight loss**	41 (13.14)	0.694 (0.513-0.937)	0.02	52 (16.67)	0.869 (0.724-1.043)	0.13	33 (10.58)	0.474 (0.285-0.788)	0.004
**Vomiting**	26 (12.15)	0.638 (0.388-1.049)	0.08	31 (14.49)	0.732 (0.399-1.342)	0.31	34 (15.89)	0.791 (0.427-1.465)	0.46
**Bloating**	66 (15.90)	0.881 (0.532-1.460)	0.62	84 (20.24)	1.137 (0.712-1.814)	0.59	57 (13.73)	0.643 (0.452-0.915)	0.01
**Abdominal pain**	218 (21.27)	1.460 (1.243-1.716)	<0.01	243 (23.71)	1.628 (1.264-2.096)	<0.001	208 (20.29)	1.125 (0.963-1.314)	0.14
**Anorexia**	49 (19.44)	1.157 (0.849-1.576)	0.36	48 (19.05)	1.039 (0.917-1.177)	0.55	38 (15.08)	0.738 (0.493-1.105)	0.14
**Constipation**	77 (19.44)	1.166 (0.942-1.443)	0.16	85 (21.46)	1.238 (0.997-1.535)	0.05	90 (22.73)	1.294 (0.991-1.689)	0.06
**Neurological symptoms**	78 (21.49)	1.353 (1.029-1.777)	0.03	77 (21.21)	1.217 (1.007-1.471)	0.04	71 (19.56)	1.039 (0.670-1.610)	0.87

© 2024 Bianchi PI et al. *JAMA Network Open.*

**Dental enamel defects**	13 (22.81)	1.401 (0.752-2.612)	0.29	12 (21.5)	1.173 (0.498-2.762)	0.71	11 (19.30)	1.011 (0.652-1.567)	0.96
**Dysproteinemia**	5 (9.09)	0.469 (0.132-1.669)	0.24	6 (10.91)	0.537 (0.150-1.921)	0.34	3 (5.45)	0.242 (0.039-1.508)	0.13
**Fatigue**	82 (18.81)	1.114 (0.702-1.766)	0.65	91 (20.87)	1.191 (0.934-1.519)	0.16	71 (16.28)	0.802 (0.570-1.129)	0.21
**Osteopenia**	6 (13.64)	0.724 (0.496-1.056)	0.09	8 (18.18)	0.965 (0.541-1.721)	0.90	7 (15.91)	0.762 (0.300-1.931)	0.57
**Failure-to-thrive**	215 (22.58)	1.619 (1.309-2.002)	<0.001	246 (25.84)	1.921 (1.706-2.162)	<0.001	175 (18.38)	0.937 (0.699-1.313)	0.71
**Marsh 0-1-2**	31 (18.45)	1.000	0.80	27 (16.07)	1.000	0.25	44 (26.19)	1.000	0.63
**Marsh 3A-3B-3C**	350 (19.43)	1.066 (0.646-1.759)		335 (18.60)	1.193 (0.884-1.610)		398 (22.10)	0.799 (0.323-1.980)	
**TTG title < 10x ULN**	209 (21.91)	1.000	0.07	177 (18.55)	1.000	0.98	254 (26.62)	1.000	0.03
**TTG title ≥ 10x ULN**	333 (15.56)	0.657 (0.420-1.028)		396 (18.50)	0.997 (0.735-1.351)		342 (15.98)	0.524 (0.293-0.938)	
**Clinical classification**									
Major	294 (18.97)	1.000		353 (22.77)	1.000		248 (16.00)	1.000	
Minor	186 (19.00)	1.002 (0.662-1.516)	0.99	178 (18.18)	0.754 (0.543-1.046)	0.09	228 (23.29)	1.594 (1.026-2.477)	0.04
Asymptomatic	45 (8.86)	0.415 (0.225-0.768)	0.005	33 (6.50)	0.236 (0.113-0.492)	<0.001	90 (17.72)	1.130 (0.557-2.296)	0.74
Potential	19 (19.39)	1.027 (0.382-2.762)	0.96	15 (15.31)	0.613 (0.243-0.373)	0.30	24 (24.49)	1.703 (0.568-5.106)	0.34
**Physician who made**									

© 2024 Bianchi PI et al. *JAMA Network Open.*

**the diagnosis**									
General pediatrician	168 (16.60)	1.000		181 (17.89)	1.000		207 (20.45)	1.000	
Hospital pediatrician	281 (16.85)	1.018 (0.595-1.740)	0.95	281 (16.85)	0.930 (0.677-1.277)	0.66	328 (19.66)	0952 (0.481-1.884)	0.89
Gastroenterologist	78 (23.49)	1.543 (0.972-2.450)	0.07	86 (25.90)	1.605 (1.199-2.149)	0.001	42 (12.65)	0.563 (0.211-1.502)	0.25
Other specialist	26 (16.35)	0.982 (0.553-1.744)	0.95	39 (24.53)	1.492 (1.072-2.078)	0.018	27 (16.98)	0.795 (0.343-1.846)	0.59

Abbreviations: CD, celiac disease; CI, confidence interval; DD, diagnostic delay; GERD, gastro-esophageal reflux disease; GI, gastrointestinal; N, number; OR, odds ratio; TTG, anti-tissue transglutaminase antibodies; ULN, upper limit of normal.

© 2024 Bianchi PI et al. *JAMA Network Open.*

eTable 4. Univariable and Multivariable Analyses for Factors Correlated to Misdiagnosis Previous to Celiac Disease Diagnosis

	**No misdiagnosis, n (%)**	**Misdiagnosis, n (%)**	**Univariable analysis OR (95% CI)**	**p-value**	**Multivariable analysis OR (95% CI)<br>Area under ROC curve 0.7450 (95%CI 0.7242-0.7650)**	**p-value**
**Total**	2958 (95.98)	124 (4.02)				
**Sex**				0.15		
**Males**	1076 (95.39)	52 (4.61)	1.000			
**Females**	1878 (96.31)	72 (3.69)	0.793 (0.579-1.086)			
**Age at diagnosis (years)**				0.54		
**<3**	607 (96.81)	20 (3.19)	1.000			
**3-5**	902 (95.75)	40 (4.25)	1.346 (0.847-2.138)	0.21		
**6-10**	977 (96.07)	40 (3.93)	1.243 (0.537-2.878)	0.61		
**11-18**	472 (95.16)	24 (4.84)	1.543 (0.694-3.432)	0.29		
**Family history of CD**	746 (97.26)	21 (2.74)	0.602 (0.371-0.977)	0.040	0.762 (0.452-1.286)	0.31
**Multiple symptoms**	812 (93.33)	58 (6.67)	2.323 (1.196-4.509)	0.013		
**Multiple diagnostic clues**	207 (97.18)	6 (2.82)	0.719 (0.495-1.046)	0.09		
**Multiple GI symptoms**	797 (93.99)	51 (6.01)	1.894 (0.796-4.506)	0.15		
**Blood cell count alterations**	674 (94.00)	43 (6.00)	1.809 (0.826-3.962)	0.14	2.118 (0.954-4.700)	0.07
**Dyspepsia**	78 (90.70)	8 (9.30)	2.546 (0.731-8.873)	0.14	2.893 (0.951-8.794)	0.06
**GERD**	27 (84.38)	5 (15.63)	4.561 (2.078-10.010)	<0.001	3.907 (1.955-7.811)	<0.001
**Diarrhea**	620 (94.66)	35 (5.34)	1.483 (0.983-2.237)	0.06	2.031 (1.222-3.376)	0.006
**Weight loss**	284 (95.95)	12 (4.05)	1.009 (0.621-1.639)	0.97		
**Vomiting**	197 (95.63)	9 (4.37)	1.097 (0.567-2.122)	0.78		
**Bloating**	370 (93.67)	25 (6.33)	1.766 (1.079-2.893)	0.02	1.439 (1.080-1.918)	0.01
**Abdominal pain**	942 (94.67)	53 (5.33)	1.598 (1.211-2.107)	0.001	1.455 (1.086-1.949)	0.01
**Anorexia**	225 (95.74)	10 (4.26)	1.065 (0.496-2.288)	0.87		
**Constipation**	353 (92.41)	29 (7.59)	2.253 (0.795-6.387)	0.13	4.659 (2.148-10.103)	<0.001
**Neurological symptoms**	325 (92.07)	28 (7.93)	2.356 (1.567-3.542)	<0.001	1.301 (0.879-1.925)	0.19
**Dental enamel defects**	48 (90.57)	5 (9.43)	2.518 (1.031-6.152)	0.043	1.189 (0.300-4.707)	0.81
**Dysproteinemia**	50 (94.34)	3 (5.66)	1.444 (0.553-3.768)	0.45		
**Fatigue**	401 (93.26)	29 (6.74)	1.943 (1.351-2.796)	<0.001	1.964 (1.515-2.545)	<0.001
**Osteopenia**	39 (97.50)	1 (2.50)	0.578 (0.261-1.281)	0.18	0.511 (0.238-1.096)	0.08

© 2024 Bianchi PI et al. *JAMA Network Open.*

**Failure-to-thrive**	892 (96.33)	34 (3.67)	0.875 (0.620-1.233)	0.45		
**Marsh-Oberhüber pre-atrophic histological lesions 0-1-2**	160 (97.56)	4 (2.44)	1.000			
**Marsh-Oberhüber atrophic lesions 3A-3B-3C**	1682 (95.68)	76 (4.32)	1.807 (1.114-2.856)	0.11	1.667 (1.147-2.424)	0.007
**TTG title < 10x ULN**	901 (97.09)	27 (2.91)	1.000			
**TTG title ≥ 10x ULN**	1991 (95.54)	93 (4.46)	1.559 (1.150-2.112)	0.004		

Abbreviations: CD, celiac disease; CI, confidence interval; GERD, gastro-esophageal reflux disease; GI, gastrointestinal; N, number; OR, odds ratio; TTG, anti-tissue transglutaminase antibodies; ULN, upper limit of normal.

© 2024 Bianchi PI et al. *JAMA Network Open.*
