# Supplementary material for: Diagnostic Delay of Celiac Disease in Childhood
Source: JAMA Netw Open. 2024 Apr 9;7(4):e245671. doi: 10.1001/jamanetworkopen.2024.5671 (PMC11004829; doi:10.1001/jamanetworkopen.2024.5671)
Supplement: Supplement 2. — Data Sharing Statement [file jamanetwopen-e245671-s002.pdf]

Data Sharing Statement

Bianchi. Diagnostic Delay of Celiac Disease in Childhood. *JAMA Netw Open*. Published April 09, 2024. doi:10.1001/jamanetworkopen.2024.5671

Data
Data available: No
